# Lymphoedema management to prevent acute dermatolymphangioadenitis in podoconiosis in northern Ethiopia (GoLBeT): a pragmatic randomised controlled trial

**DOI:** 10.1016/S2214-109X(18)30124-4

**Published:** 2018-05-15

**Authors:** Henok Negussie, Meseret Molla, Moses Ngari, James A Berkley, Esther Kivaya, Patricia Njuguna, Greg Fegan, Abreham Tamiru, Abebe Kelemework, Trudie Lang, Melanie J Newport, Andy McKay, Fikre Enquoselassie, Gail Davey

**Affiliations:** a Wellcome Trust Centre for Global Health Research, Brighton and Sussex Medical School, Brighton, UK; b Centre for Environmental and Developmental Studies, Addis Ababa University, Addis Ababa, Ethiopia; c School of Public Health, Addis Ababa University, Addis Ababa, Ethiopia; d KEMRI Wellcome Trust Research Programme, Kilifi, Kenya; e Swansea University Medical School, Swansea, UK; f International Orthodox Christian Charities, Debre Markos, Ethiopia; g Centre for Tropical Medicine and Global Health, University of Oxford, Oxford, UK; h Department of Economics, University of Sussex, Brighton, UK

## Abstract

**Background:**

Podoconiosis (also known as endemic, non-filarial elephantiasis) affects about 4 million subsistence farmers in tropical Africa. Poor awareness of the condition and inadequate evidence for the efficacy of treatment mean that no government in an endemic country yet offers lymphoedema management for patients with podoconiosis. Among patients with filarial lymphoedema, trials suggest that limb care is effective in reducing the most disabling sequelae: episodes of acute dermatolymphangioadenitis. We aimed to test the hypothesis that a simple, inexpensive lymphoedema management package would reduce the incidence of acute dermatolymphangioadenitis in adult patients with podoconiosis in northern Ethiopia.

**Methods:**

We did a pragmatic randomised controlled trial at health posts and health centres in 18 sub-districts of Aneded woreda (district) in Amhara, northern Ethiopia. Participants were adults aged 18 years and older, had a diagnosis of at least stage 2 podoconiosis (persistent lymphoedema) and a negative antigen test for filariasis, and intended to remain within Aneded woreda for the duration of the trial. Patients were randomly assigned (1:1) to either receive a package containing instructions for foot hygiene, skin care, bandaging, exercises, and use of socks and shoes, with support by lay Community Podoconiosis Agents at monthly meetings (intervention group) or to receive no intervention (control group). Participants were aware of their group assignment, but researchers doing all analyses were masked to treatment group. The primary outcome was incidence of acute dermatolymphangioadenitis episodes in the total period of observation of each participant, measured by use of validated patient self-reported pictorial diaries. This trial was registered with the International Standard Randomised Controlled Trials Number Register, number ISRCTN67805210.

**Findings:**

Between Dec 1, 2014, and June 30, 2015, 1339 patients were screened, and 696 patients were enrolled and randomly allocated to treatment groups. We allocated 350 patients to the intervention group and 346 patients to the control group. 321 (92%) patients from the intervention group and 329 (95%) patients from the control group provided follow-up results at 12 months. During the 12 months of follow-up, 16 550 new episodes of acute dermatolymphangioadenitis occurred during 765·2 person-years. The incidence of acute dermatolymphangioadenitis was 19·4 episodes per person-year (95% CI 18·9–19·9) in the intervention group and 23·9 episodes per person-year (23·4–24·4) in the control group. The ratio of incidence rate in the intervention group to that of the control group was 0·81 (0·74 to 0·89; p<0·0001), with a rate difference of −4·5 (−5·1 to −3·8) episodes per person-year. No serious adverse events related to the intervention were reported.

**Interpretation:**

A simple, inexpensive package of lymphoedema self-care is effective in reducing the frequency and duration of acute dermatolymphangioadenitis. We recommend its implementation by the governments of endemic countries.

**Funding:**

Joint Global Health Trials scheme (from the Wellcome Trust, the UK Medical Research Council, and UK Aid).

## Introduction

Podoconiosis is a form of lymphoedema that arises in tropical highland areas in genetically susceptible people who do not use footwear.^1^, ^2^ This disease is an important problem in tropical Africa, where irritant volcanic soils that are rich in silicates have been generated by the environmental conditions of high altitude (>1000 m) and high rainfall (>1000 mm per year), and these soils are farmed by very poor people who cannot afford shoes or water for washing.^3^ In affected individuals, mineral particles penetrate the skin, which is characterised by low hydration of the stratum corneum,^4^ and these particles are taken up by macrophages in the dermis, afferent lymphatic vessels, and lymph nodes. The primary lymphatic vessels become dilated and surrounded by lymphocytes as oedema and disorganised collagen production occurs. If fibrosis predominates, the lymphatic lumen narrows and is eventually destroyed; if oedema predominates, lymphatic valvular dysfunction tends to occur.^5^

**Research in context**

**Evidence before this study**

We did a search of PubMed and Google with the search terms “podoconiosis” and “non-filarial elephantiasis”, and we manually searched the reference lists of articles identified. The search was from the beginning of time until April 20, 2016, and included all articles in English, French, and Spanish. Two studies on management of podoconiosis lymphoedema have been done. The first of these studies was an uncontrolled follow-up study of 30 patients with podoconiosis for 1 year, which found reductions in disease stage and leg circumference and increased quality of life associated with a programme comprising foot hygiene, bandaging, and use of socks and shoes. The second study was a randomised trial that used 2% glycerine in the soaking water, with a primary outcome of skin barrier function. Neither of these studies assessed the incidence of acute dermatolymphangioadenitis. Several observational studies of patients with filarial lymphoedema have suggested the value of hygiene treatment in prevention of acute dermatolymphangioadenitis but, to our knowledge, no trial had been done.

**Added value of this study**

To our knowledge, this is the first randomised controlled trial to measure the effects of a lymphoedema management package on the most important consequence of podoconiosis, acute attacks of dermatolymphangioadenitis. Our study showed that pragmatic trials can be done in challenging, low-resource settings with high rates of follow-up, and that acute attacks are more frequent among untreated patients than previously thought.

**Implications of all the available evidence**

Simple limb hygiene and foot care measures are effective in preventing acute dermatolymphangioadenitis in podoconiosis lymphoedema. These findings are likely to inform guidelines for elimination of podoconiosis in endemic countries.

Although rarely a direct cause of mortality, podoconiosis disables an estimated 4 million subsistence farmers in tropical Africa; this disease reduces productivity^6^ and leads to substantial stigmatisation by the community^7^, ^8^, ^9^ and by health professionals,^10^ low quality of life,^11^ and depression.^12^ Episodes of acute dermatolymphangioadenitis are among the most severe clinical consequences of lymphoedema. The causes of acute dermatolymphangioadenitis among patients with podoconiosis are likely to be similar to its causes among patients with other types of lymphoedema, such as filarial or post-surgical lymphoedema. Episodes of acute dermatolymphangioadenitis are characterised by malaise, fever, chills, diffuse inflammation, swelling of the limbs, lymphangitis, adenitis and, eventually, skin peeling.^13^ These episodes are known locally as “michader”. They occur frequently (reports vary from five to 23 episodes per year), and contribute substantially to the disability and social effects associated with podoconiosis.^12^, ^14^, ^15^, ^16^ Antibiotic therapy (including penicillin), which has been recommended for interruption of acute dermatolymphangioadenitis episodes,^17^ is not routinely available for patients in remote rural populations in Ethiopia. Despite the high impact of podoconiosis on rural farming communities, treatment and control of podoconiosis have been hampered by misdiagnosis (chiefly, confusion with filarial lymphoedema) and fatalism.^10^

In Ethiopia, where 1·5 million people are affected by podoconiosis,^18^ foot care has been offered to people with podoconiosis through small non-government organisations in some areas of the three most heavily affected regions: Amhara, Oromia, and Southern Nations Nationalities and Peoples regions. This local, so-called bottom-up innovation^19^ has developed from the grassroots level with little formal evaluation. To date, two small studies^20^, ^21^ have assessed foot hygiene in podoconiosis: an uncontrolled follow-up study^20^ showed clinical improvements and improvements to quality of life associated with food hygiene practice and a trial^21^ that used small volumes of water with glycerol showed equivalent effects on skin barrier function to treatment with larger volumes of water. Evidence of the effects of lymphoedema management on prevention of acute dermatolymphangioadenitis, the most disabling consequence of podoconiosis lymphoedema, is unavailable. Thus, there is an urgent need for evidence on which to base national and international policy.

Among individuals with filarial lymphoedema, observational studies^22^, ^23^, ^24^ have shown that lymphoedema care is effective in reducing the incidence of acute dermatolymphangioadenitis, disease stage, and disability. To our knowledge, evaluation of similar care on the incidence of acute dermatolymphangioadenitis in podoconiosis lymphoedema has not been done. In GoLBeT (the Gojjam Lymphoedema Best Practice Trial), we tested the hypothesis that a simple lymphoedema treatment package (comprising information about foot hygiene, skin care, bandaging, exercises to improve lymph drainage, and use of socks and shoes) would reduce the incidence of acute dermatolymphangioadenitis.

## Methods

### Study design and participants

We did a pragmatic, randomised controlled trial at health posts and health centres in 19 kebeles (subdistricts) of Aneded woreda (district) in the Amhara region of northern Ethiopia. The trial protocol has been published previously.^25^ Participants were adults aged 18 years or older, had a diagnosis of at least stage 2 podoconiosis (persistent lymphoedema)^26^ and a negative BinaxNOW Filariasis rapid antigen test (Alere Inc, Waltham, MA, USA), and intended to remain within Aneded woreda for the duration of the trial. These participants were identified by Health Extension Workers, who listed potential patients.^27^ Participants were excluded if they had a mental or physical disability or disease that would impair their ability to adhere to the intervention, such as a nodular disease preventing use of shoes. We intended for these patients to be representative of the population of people with podoconiosis in Amhara, northern Ethiopia, where the prevalence of podoconiosis in adults was estimated to be 3·9% in the 2013 nationwide mapping.^28^

The trial was done in accordance with the Helsinki Declaration and the International Conference on Harmonisation Good Clinical Practice guidelines and was approved by the Institutional Review Board of the College of Health Sciences, Addis Ababa University (071/13/SPH), the National Ethical Review Committee of the Ethiopian Science and Technology Agency (3-1/794/06), the Food, Medicine and Health Care Administration Authority of Ethiopia (02/6-1/05/39), and the Research Ethics and Governance Committee of Brighton and Sussex Medical School (13/107/DAV). Participants gave informed consent and either signed the consent form themselves or, if unable to write, added thumbprints that were countersigned by an independent witness.

### Randomisation and masking

We randomly allocated patients (at a 1:1 ratio) to either the intervention group (who received instructions for foot hygiene, skin care, bandaging, exercises, and use of socks and shoes, and support by lay Community Podoconiosis Agents at monthly meetings) or the control group (who received no intervention). Computer-based individual randomisation was done at the KEMRI Wellcome Trust Research Programme Clinical Trials Facility in Kilifi, Kenya, by use of enrolment lists sent by the trial data manager (MM) to the trial statistician (GF; who had no other involvement in the trial). This randomisation was done by use of the ralloc command in STATA version 14. The process of assignment to treatment groups was masked from the statisticians performing the analyses (MN and JAB), but an individual patient's group status could not be masked to the field team and participants were aware of their allocation. However, the researchers doing all analyses were masked to treatment groups.

### Procedures

The intervention consisted of monthly group meetings with instruction and practical demonstration of lymphoedema management by Community Podoconiosis Assistants (locally appointed women, who were given 2 weeks of training), to support daily self-treatment. Lymphoedema management comprised foot hygiene (soaking the feet, washing with bathroom soap with a pH lower than 9 to minimise the effects of this soap on skin barrier function, rinsing with clean water, drying, and applying emollient) and foot care (supervised use of single-layer, non-elastic bandages for disease stages of at least 3; foot and calf exercises; instruction to practise foot hygiene daily at home, to elevate the foot of the bed or sleeping area, and to use socks and shoes during waking hours; provision of shoes and socks at month 3, when the most easily reversible oedema had subsided). Patients in the control group were told that they were on a waiting list for treatment, to start 12 months later, and were not given the instructions or demonstrations that the intervention group received. Scheduled follow-up visits for data collection were made 3, 6, 9, and 12 months after study initiation by data collectors who were independent of the Community Podoconiosis Assistants. Patients in the control group were visited on different days and at different sites to those in the intervention group, to minimise spillover.

Patients in both groups were trained to record episodes of acute dermatolymphangioadenitis in monthly pictorial diaries that were specifically developed for the study and validated against assessment by health professionals. The feasibility of diary completion and the validity of self-reports of acute dermatolymphangioadenitis against diagnosis by health workers had also been checked in a small pilot study before the trial.^29^ The positive predictive value of patients' self-reports of episodes of acute dermatolymphangioadenitis was 0·87.

### Outcomes

The primary outcome was cumulative incidence of episodes of acute dermatolymphangioadenitis in all patients with available data from treatment initiation until up to 12 months of follow-up, compared between intervention and control groups. Secondary outcomes were foot care behaviours (foot washing, use of ointment, use of bandages, elevation, exercises, use of socks and shoes), measured by questionnaire of both groups at baseline and 12 months; clinical outcomes (clinical stage of disease, lower leg and foot circumferences, presence of mossy changes, wounds and interdigital lesions, duration of acute dermatolymphangioadenitis days); social outcomes (quality of life, perceived stigma); and economic productivity. Social and economic outcomes will be reported in a future publication. The outcomes recorded at each visit are shown in the appendix; prespecified interactions between age, sex, and kebele location and the main effects were calculated with likelihood ratio tests. Adverse events were reported on the case record forms completed by the data collectors at the quarterly home visits.

During the trial team's pre-study visit, the staff providing treatment in nearby districts reported that affected people rarely adopted the lymphoedema management package, even if they saw it in use in their village. These reports persuaded us to use individual rather than cluster randomisation, although we decided not to randomise more than one patient per household. Given that patients within the same subdistricts could be allocated to different groups, we also examined for possible use of the intervention measures in the control group through reported behavioural practices at baseline and month 12 in case report forms.

### Statistical analysis

We estimated that 340 participants in each group would give 90% power to detect a reduction in frequency of acute dermatolymphangioadenitis episodes of 28% (from 5·6 to 4·0 episodes per year), given a mean baseline incidence of acute dermatolymphangioadenitis of 5·6 (SD 4·9) episodes per year,^16^ an α of 0·05, an ability to examine the effects of up to four confounders, and 15% dropout.^20^ A statistical analysis plan was reviewed and agreed by the Trial Steering Committee before locking and unmasking the database. No interim analysis was planned or done. We analysed the primary outcome in all participants assigned to the intervention or control groups who completed diaries at 3, 6, 9, and 12 months. The incidence of new acute dermatolymphangioadenitis episodes was defined as the number of episodes, divided by the total duration of observation for each participant. We examined whether treatment outcome differed by sex, by disease severity at baseline, or by kebele location. For kebele location, we created two groups: close (nine kebeles) and remote (nine kebeles), and tested for group effect modification on the study outcome with a likelihood ratio test. We calculated the duration of observation for each participant until the trial conclusion, death, or dropout. We compared the incidence ratios of patients in the intervention group with those in the control group using Poisson regression with robust standard errors, to allow for clustering of events within participants. The duration of episodes of acute dermatolymphangioadenitis was computed by adding the duration of all episodes in days and dividing by the number of days of observation for each participant, and these durations were compared with a rank sum test. Other secondary outcomes will be reported in a future publication. Analysis was done by an intention-to-treat protocol. STATA was used for all statistical analyses. No Data Safety and Monitoring Committee was needed, since the intervention is publicly available, commonly used, and safe. The trial is registered with the International Standard Randomised Controlled Trials Number Register, number ISRCTN67805210.

### Role of the funding source

The funders of the study had no role in data collection, data analysis, data interpretation, or writing of the report. The study design was altered (from a stepped-wedge design to an individually randomised trial) before funding of the proposal, in accordance with comments from reviewers who acted on behalf of the joint funders. The corresponding author had full access to all the data in the study and had final responsibility for the decision to submit for publication.

## Results

Between Dec 1, 2014, and June 30, 2015, 1339 patients with podoconiosis were screened for eligibility; 696 patients were enrolled and 643 were excluded (figure). 350 study participants were randomly assigned to the intervention group and 346 to the control group. 46 (7%) patients did not complete the study. 29 patients (4% of all patients enrolled; 63% of non-completers) dropped out in the intervention group and 17 (2%; 37%) dropped out in the control group (p=0·07). At the end of the study (after 12 months), 321 (92%) of 350 patients in the intervention group and 329 (95%) of 346 patients in the control group remained in the trial (figure).

**Figure:**
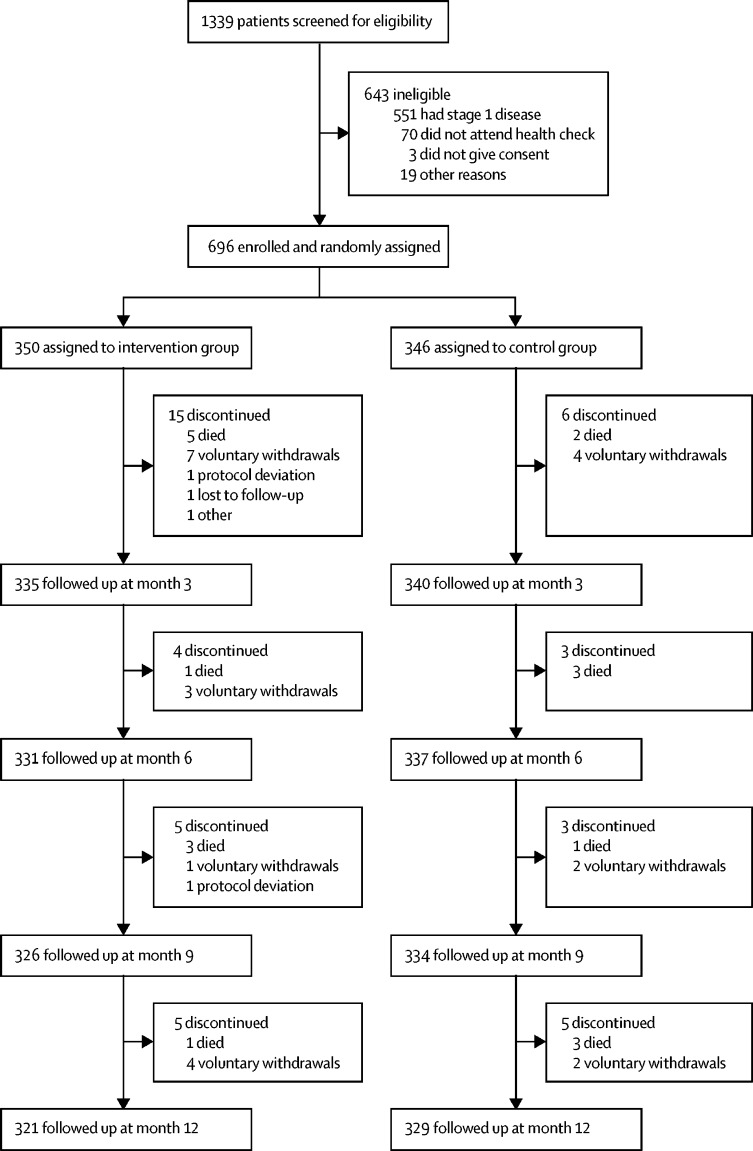
Trial profile

Table 1 shows the baseline characteristics of the study groups. The median age (IQR) of the study participants was 50 (40–61) years. Only 19% of participants had any formal education. WHO Disability Assessment Schedule-II scores,^30^ total stigma scores on the validated podoconiosis stigma scale,^31^ Dermatology Quality of Life Index scores,^11^ and clinical measures (episodes of acute dermatolymphangioadenitis in the previous month, disease stage, presence of mossy lesions, and lower leg and foot circumferences) were similar between groups.

**Table 1:** Baseline characteristics

	**Intervention group (n=350)**	**Control group (n=346)**
Age, years	50 (40–60)	52 (41–65)
Women	172 (49%)	164 (47%)
Married	226 (65%)	226 (65%)
Ever attended school	66 (19%)	66 (19%)
Asset index quintile 1*	77 (22%)	75 (22%)
Own shoes	219 (63%)	231 (67%)
Own socks	76 (22%)	64 (18%)
Use soap to wash their feet	198 (57%)	186 (54%)
Use ointment on their feet	110 (31%)	128 (37%)
Ever had acute dermatolymphangioadenitis	341 (97%)	341 (99%)
Number of acute dermatolymphangioadenitis episodes in past 30 days	2 (2–3)	3 (2–3)
Average duration of the latest episode, days	3 (3–5)	3 (3–5)
Mossy lesions present on either leg	184 (53%)	185 (53%)
Interdigital lesions present on either leg	89 (25%)	95 (27%)
Right lower leg circumference, cm	23 (21–27)	24 (22–27)
Left lower leg circumference, cm	24 (22–26)	24 (22–26)
Left foot circumference, cm	25 (24–27)	25 (24–27)
Right foot circumference, cm	25 (24–27)	25 (24–27)
Total WHO Disability Assessment Schedule II score^30^	35 (28–39)	34 (25–39)
Total stigma score^31^	18 (12–21)	17 (12–21)
Total Dermatology Quality of Life Index^11^	21 (14–32)	21 (14–31)

Data are n (%) or median (IQR).

* Asset index derived by use of principal component analysis, and these data indicate participants in the poorest 20% of the asset index.

During the 12 months of follow-up, 16 550 episodes of acute dermatolymphangioadenitis within 765·2 person-years were recorded, giving an overall incidence of 21·6 episodes per person-year (95% CI 21·3–21·9) across both groups (table 2). Overall, there were 7515 episodes in the intervention group during 387·1 person-years, and 9035 episodes in the control group during 378·1 person-years, giving an incidence of 19·4 episodes per person-year (18·9–19·9) in the intervention group and 23·9 episodes per person-year (23·4–24·4) in the control group. The incidence rate ratio of the intervention group relative to the control group was therefore 0·81 (0·74–0·89; p<0·0001), with a difference of −4·5 episodes per person-year (−5·1 to −3·8).

**Table 2:** Incidence of episodes of acute dermatolymphangioadenitis, overall and by quarter

	**Intervention group (n=350)**			**Control group (n=346)**			Incidence ratio (95% CI)*	**Incidence difference (95% CI)**
	Person-years	Number of events	Incidence† (95% CI)	Person-years	Number of events	Incidence† (95% CI)		
Overall	387·1	7515	19·4 (18·9–19·9)	378·1	9035	23·9 (23·4–24·4)	0·81 (0·74 to 0·89)	−4·5 (−5·1 to −3·8)
Q1	102·3	1227	12·0 (11·3–12·7)	102·1	1642	16·1 (15·3–16·9)	0·75 (0·63 to 0·88)	−4·1 (−5·1 to −3·1)
Q2	100·1	2492	24·9 (23·9–25·9)	100·0	2903	29·0 (27·9–30·1)	0·86 (0·77 to 0·95)	−4·1 (−5·6 to −2·7)
Q3	97·5	2182	22·4 (21·5–23·3)	96·9	2555	26·4 (25·3–27·4)	0·85 (0·78 to 0·94)	−4·0 (−5·4 to −2·6)
Q4	87·2	1614	18·5 (17·6–19·4)	79·0	1935	24·5 (23·4–25·6)	0·76 (0·66 to 0·86)	−6·0 (−7·4 to −4·6)

* Calculated by use of Poisson regression, to account for participant clustering of events.

† Episodes per person-year.

We found that the intervention had increasing efficacy in reducing the risk of acute dermatolymphangioadenitis episodes over time (p=0·0012 between the quarterly intervals; table 2). Although there was no difference in the incidence of acute dermatolymphangioadenitis episodes by sex at baseline (p=0·10), we also found greater efficacy of the intervention in men than women; women had a higher incidence of acute dermatolymphangioadenitis episodes and higher ratio of acute dermatolymphangioadenitis incidence relative to the control group than did men (both p<0·0001; table 3). Age at enrolment to the trial did not significantly modify the effect of the intervention (p=0·114; appendix). The number of participants living in kebeles close to or remote from the surfaced road did not differ between the intervention and control groups (p=0·67). However, kebele location affected intervention efficacy: the incidence rate ratio was 0·91 (95% CI 0·82 to 1·02) in participants from the nine closer kebeles and 0·59 (0·49 to 0·72) in those from the nine more remote kebeles (p=0·0003).

**Table 3:** Incidence of acute dermatolymphangioadenitis episodes and adherence, stratified by sex

		**Overall (n=350)**	**Men (n=178)**	**Women (n=172)**	**p value**
Incidence of acute dermatolymphangioadenitis episodes* (95% CI)		21·6 (21·3–21·9)	20·9 (20·4–21·3)	22·4 (21·9–22·9)	<0·0001
Ratio of acute dermatolymphangioadenitis incidence, intervention to control groups (95% CI)		0·81 (0·69–0·96)	0·73 (0·64–0·84)	0·90 (0·79–1·02)	<0·0001
Adherence					
	To overall intervention	95·4% (94·6–96·1)	95·4% (94·7–96·1)	95·3% (94·4–96·1)	0·79
	To washing with soap	98·6% (98·2–99·1)	98·5% (97·9–99·0)	98·9% (98·4–99·3)	0·27
	To applying ointment	98·0% (97·4–98·8)	97·9% (97·2–98·6)	98·2% (97·5–99·0)	0·48
	To leg elevation	97·4% (96·3–98·4)	97·7% (96·9–98·5)	97·0% (95·7–98·3)	0·34
	To exercises	97·3% (96·4–98·2)	97·6% (96·8–98·3)	97·0% (96·0–98·1)	0·40
	To wearing socks	87·2% (84·8–89·5)	87·1% (84·9–89·3)	87·2% (84·7–89·6)	0·98
	To wearing shoes	93·6% (92·4–94·9)	93·8% (92·7–95·0)	93·4% (92·1–94·7)	0·60

Data are mean proportion (95% CI), unless otherwise indicated. These data are based on adherence records collected from participants in the intervention group only, which are from reported data from the previous month (at 3, 6, 9, and 12 months).

* Episodes per person-year.

Adherence by participants to the trial intervention was consistently above 85% for washing their feet and lower legs with soap, applying ointment, use of bandages, elevation, and exercise during the 12 months of the study (table 3). However, adherence to instructions to wear socks and shoes was lower, even after the first 3 months, when custom shoes had been supplied (appendix). There was no evidence that adherence to the overall intervention (p=0·79) or any specific elements (p>0·05; table 3) varied by sex.

The duration of acute dermatolymphangioadenitis episodes ranged from 1 to 35 days, with a higher geometric mean in the control group than the intervention group (p<0·001; table 4). 2915 (17·6%) of the 16 550 acute dermatolymphangioadenitis episodes lasted for 1 day, comprising 1463 (19%) of 7515 episodes in the intervention group and 1452 (16%) of 9035 episodes in the control group (p<0·0001). The geometric mean duration of acute dermatolymphangioadenitis episodes per year was 18·9 (95% CI 18·6 to 19·2) days in the intervention group and 25·6 (95% CI 15·2 to 26·0) days in the control group (p<0·0001).

**Table 4:** Duration of acute dermatolymphangioadenitis symptoms

	**Intervention group (n=350)**	**Control group (n=346)**	**p value**
Duration of episodes of acute dermatolymphangioadenitis, geometric mean days (95% CI)	2·1 (2·0–2·4)	2·3 (2·2–2·4)	<0·0001
Total duration of symptoms of acute dermatolymphangioadenitis, median days (IQR)	96 (54–129)	116 (80–154)	<0·0001
Total duration of symptoms of acute dermatolymphangioadenitis per person-year, median days (IQR)	22 (12–30)	26 (17–36)	<0·0001

No serious adverse events were related to the intervention or intervention products. Of the 19 deaths during the trial, ten (53%) occurred in the intervention group and nine (47%) in the control group (p=0·84). Nine of the patients who died were men and ten were women.

In the analysis of potential spillover (contamination), both groups of patients reported increased frequency of washing their feet, of washing their feet with soap, and of applying ointment (table 5), although the increases in these behaviours were significantly smaller in the control group (p<0·001).

**Table 5:** Participant answers to questions on behavioural practices by intervention group

	**Intervention group (n=350)**	**Control group (n=346)**	**p value**
**Washed feet last night?**			
Baseline	307 (88%)	298 (86%)	0·54
Month 12	319 (91%)	317 (92%)	0·02*
**How many times in the past week did you wash your feet?**			
Baseline	223 (64%)	218 (63%)	0·85
Month 12	291 (83%)	277 (80%)	0·29
**How many times in the past week did you wash your feet with soap?**			
Baseline	39 (11%)	42 (12%)	0·68
Month 12	242 (69%)	140 (40%)	<0·0001
**Over the last month, have you put any ointment on your feet?**			
Baseline	111 (32%)	128 (37%)	0·16
Month 12	259 (74%)	160 (46%)	<0·0001

Group data are number of affirmative responses (%) or number that answered “at least seven times” (%). Data are based on reports by all patients at baseline and at 12 months and refer to their behaviours during the past week. p value data are from a χ^2^ test, or

* Fisher's exact test.

## Discussion

To our knowledge, GoLBeT is the first trial to assess the effects of a lymphoedema management package on the most important clinical consequence of podoconiosis lymphoedema: the incidence of acute dermatolymphangioadenitis episodes. Our trial showed a clear effect of the intervention, with an overall incidence ratio of 0·81, and evidence of cumulative benefit over time. Baseline incidence of acute dermatolymphangioadenitis and incidence rate in the control group during the trial were similar to those reported in western Ethiopia in 2016,^15^ but much higher than those reported in northern Ethiopia in 2012 (and used to calculate the sample size) for this trial,^14^ highlighting how little was previously known about this important sequela. The reduction in incidence is smaller than the reduction shown in uncontrolled prospective studies among patients with lymphatic filariasis in Haiti,^24^ Burkina Faso,^32^ and Odisha state, India,^22^ where reductions ranged from 33% to 69%. A meta-analysis^33^ of hygiene-based lymphoedema management in lymphatic filariasis showed an acute dermatolymphangioadenitis incidence ratio of 0·32 (95% CI 0·25–0·40) across the eight studies with sufficient (non-randomised) information from before and after treatment. The smaller reduction measured in our trial compared with these previous studies might reflect a different disease process, the greater stringency of a randomised controlled trial, spillover (contamination)^34^ across randomisation groups, or different methods of gathering primary outcome data. This difference in results also indicates that use of a simple package is one approach to preventing acute dermatolymphangioadenitis, but that more research is needed to identify other approaches.

The reported increased frequency of foot washing, of washing feet with soap, and of applying ointment in both groups suggest a Hawthorne effect^35^ (when simply being studied alters behaviour) that could have driven spillover. Together, these influences are likely to have reduced the observed magnitude of the difference in incidence of acute dermatolymphangioadenitis episodes between groups.

Primary outcome data were gathered through monthly self-reported diaries, which were supported by one researcher-administered question in the quarterly case report form. Previous validation of self-reports of acute dermatolymphangioadenitis episodes by use of the trial diaries gave a positive predictive value of 0·87 when compared with diagnoses by health professionals.^29^ The false-positive self-reports are likely to represent conditions that are not amenable to foot hygiene treatment, so it is possible that the actual effect of this treatment package on acute dermatolymphangioadenitis was larger than we measured. Given our concern with altering the behaviour of patients in the control group, contact with these patients (and diary collection) only occurred every 3 months, rather than monthly (as for patients in the intervention group). Diaries were always carefully checked when they were collected, but it is possible that the lower frequency of collection in the control group led to bias through fewer episodes being reported in this group.

An earlier uncontrolled follow-up of patients who were offered a similar lymphoedema management package suggested slightly greater improvement in clinical outcomes among men than women.^20^ This trial was therefore powered to enable subanalysis by sex; we also found a significantly greater effect of this package among men (20·9 episodes per person-year among men *vs* 22·4 episodes per person-year among women). We found no difference in frequency of baseline acute dermatolymphangioadenitis episodes by sex (p=0·10; data not shown), and there was no evidence of any difference in adherence overall or to specific intervention components by sex. To our knowledge, studies^33^ of lymphoedema management in patients with lymphatic filariasis have not disaggregated data by sex so it is difficult to contextualise this finding. Observational studies^14^, ^36^ among people with podoconiosis suggest that men have more access to shoes, and are more likely to own a pair. In the general population, men are more likely to be found wearing shoes at interview.^37^ However, sex-related differences in access to foot hygiene treatment have not been documented.^38^ The most robust studies^37^ on podoconiosis prevalence showed that more women than men were affected in Ethiopia. Our findings underscore the importance of ensuring gender equity in provision, uptake, and effects of lymphoedema management services.

We found evidence of effect modification by location: participants in more remote kebeles recorded a greater effect of the treatment package than those in kebeles closer to the surfaced road. This finding could not be explained by differences in baseline disease severity (remote *vs* close kebeles*:* p=0·19, left leg disease stage; p=0·39, right leg disease stage) or access to water for washing (p=0·09). We anticipate that the results of our qualitative process assessment could enable us to better interpret this interaction.

We have found clinically important and significant reductions in incidence of acute dermatolymphangioadenitis episodes, episode duration, and days with acute dermatolymphangioadenitis symptoms after administration of a lymphoedema management package compared with no intervention. In the intervention group, the proportion of acute attacks that lasted only a single day was significantly higher, and the total duration of acute attacks was significantly lower than in the control group (table 4). These findings suggest that the foot hygiene package might shorten attacks and prevent their occurrence. In two studies^39^, ^40^ of patients with lymphatic filariasis, duration of acute dermatolymphangioadenitis also decreased with foot care, and the magnitude of the change was similar to our findings among people with podoconiosis lymphoedema. Shorter duration of acute dermatolymphangioadenitis might have also been reflected in the number of days in the past month during which patients were unable to carry out their usual activities, which was significantly fewer in the intervention group.

GoLBeT achieved a very high proportion of patients who completed follow-up (92% in the intervention group, 95% in the control group) given the remote setting of many of the kebeles and difficulties in seasonal access. This proportion was considerably higher than that assumed when calculating the necessary sample size (85%), and seems to have been the consequence of employing retention strategies (as advised in the Rapid Ethical Assessment;^41^ for example, patients in the control group were given small packs of coffee at data collection visits), relationship-building between patients and the trial team, and determined fieldwork.

Given the nature of the intervention, full masking was not possible in this trial. Patients were aware of their randomisation status. The duties and responsibilities of data collectors were independent of those delivering treatment, but they might have been able to guess the participant's group if they saw the supplies required for treatment.

The trial setting meant that, although adverse events were reported at each data collection visit, passive reporting was slow and some adverse events might have been missed. Although patients, their families, Community Podoconiosis Assistants, and data collectors were asked to report adverse events immediately, immediate reporting was difficult in practice. Patients and relatives were also asked to notify the receiving doctor of their participation in the trial if they were an outpatient or inpatient at the district hospital. Receiving doctors were given an information session about the trial and asked to contact the trial coordinator immediately if a participant consulted them. The number of days between death and report reaching the Trial Coordinator ranged from less than 1 day to 28 days (for one incident). Once reported to the trial team, all adverse events were reported on the same day to the Local Safety Monitors by telephone and were followed up with an email when the serious adverse event form had been completed. Although no serious adverse events were considered to be related to the intervention products, it is possible that we missed some minor adverse events.

GoLBeT was pragmatic in terms of the setting in which it was done: patients were offered treatment in their communities, which were remote, and vehicle access was often difficult. The intervention took the form of supported self-management; daily self-care was supported by monthly group meetings that reinforced technique and replenished treatment supplies. Our trial is a good example of rigorous evaluation of a frugal innovation—ie, a low-tech approach to solve unmet local needs.^19^ Although we have shown that this management package is effective, wider adoption by government and non-government stakeholders depends not only on effectiveness, but also on cost, acceptability, and sustainability. We anticipate that a process evaluation done during the closing stages of this trial will shed further light on acceptability, feasibility, and sustainability, and cost-effectiveness will be presented separately after further analyses are completed. The message for national policy makers is clear: adoption of a simple lymphoedema management package is effective against incidence and duration of acute dermatolymphangioadenitis, the most disabling consequence of podoconiosis.
