# Supplementary material for: Lymphoedema management to prevent acute dermatolymphangioadenitis in podoconiosis in northern Ethiopia (GoLBeT): a pragmatic randomised controlled trial
Source: Lancet Glob Health. 2018 May 15;6(7):e795–803. doi: 10.1016/S2214-109X(18)30124-4 (PMC6562300; doi:10.1016/S2214-109X(18)30124-4)
Supplement: Supplementary appendix [file mmc1.pdf]

THE LANCET
Global Health

## Supplementary appendix

This appendix formed part of the original submission and has been peer reviewed. We post it as supplied by the authors.

Supplement to: Negussie H, Molla M, Ngari M, et al. Lymphoedema management to prevent acute dermatolymphangioadenitis in podoconiosis in northern Ethiopia (GoLBeT): a pragmatic randomised controlled trial. *Lancet Glob Health* 2018; published online May 14. http://dx.doi.org/10.1016/S2214-109X(18)30124-4.

**Lymphoedema management to prevent acute dermatolymphangioadenitis in podoconiosis (GoLBeT): a pragmatic randomised controlled trial in northern Ethiopia**

**Supplementary Appendix**

Henok Negussie[1]*, Meseret Molla[2]*, Moses Ngari[3], James A Berkley[3, 6], Esther Kivaya[3], Patricia Njuguna[3], Greg Fegan[3,4], Abreham Tamiru[5], Abebe Kelemework[5], Trudie Lang[3,6], Melanie J Newport[1], Andy McKay[7], Fikre Enquoselassie[8], Gail Davey[1,8†].

*joint first authors; †corresponding author.

Affiliations.

1. Wellcome Trust Centre for Global Health Research, Brighton & Sussex Medical School, Brighton, UK.
2. Centre for Environmental and Developmental Studies, Addis Ababa University, Addis Ababa, Ethiopia.
3. KEMRI/Wellcome Trust Research Programme, Kilifi, Kenya.
4. Swansea University Medical School, Swansea, Wales.
5. International Orthodox Christian Charities Podoconiosis Project, Debre Markos, Ethiopia
6. Centre for Tropical Medicine & Global Health, University of Oxford, UK.
7. Department of Economics, University of Sussex, UK.
8. School of Public Health, Addis Ababa University, Addis Ababa, Ethiopia.

## Supplementary Methods

*Study Participants.* Patients were excluded if they had nodular disease preventing the use of custom shoes; complex wounds; a history of allergic reaction to Whitfield ointment; a physical disability, serious mental health problem or other condition that would limit self-treatment; or if they were already undertaking self-treatment.

*Study Location.* The trial team was based in Debre Markos, a town approximately 15k from Aneded *woreda* and the site of the first podoconiosis treatment project in Amhara Region, established by International Orthodox Christian Charities. At the time of this trial, no government or private treatment for podoconiosis was available within Aneded *woreda*.

*Enrolment.* Following the listing of people known to have leg swelling by Health Extension Workers (female health promotion workers deployed two per *kebele* throughout Ethiopia), potential participants were visited at home. Initial oral consent was sought for screening of those who fulfilled inclusion and exclusion criteria at this visit (age, intention to stay in *woreda*, no current self-treatment). Patients who then attended an enrolment appointment at a nearby health post were given further information about the trial (taking into account the community's expressed preferences for content and delivery of information elicited during Rapid Ethical Assessment [1]). Individual informed consent was provided, either signed by the participant or thumbprinted and then signed by an independent witness, as agreed by the Institutional Review Boards (IRBs). Eligibility criteria, consent, serious adverse events and study endpoints were verified against source documents for all participants by visiting monitors.

*Randomisation.* Enrolment was first conducted in the nine most accessible *kebeles* in Aneded *woreda* (*kebeles* 7 to 14 and 16 in Figure 1), yielding a list of 401 potential participants (Round 1). These participants were allocated consecutive randomised study numbers with gaps of 5 numbers left between *kebeles*, and intervention started in those randomised to the intervention group. Meanwhile enrolment continued in these *kebeles* plus a further nine less accessible *kebeles*, (1-6 , 15, 17 and 18 in Figure 1) yielding a second list of 295 potential participants (Round 2) who were randomised in the same way as Round 1.

*Intervention.* Those randomised to the intervention group received the intervention for one year starting within 1 month of randomisation, while those randomised to the control group were offered the intervention for one year starting 13 months after randomisation.

*Study Endpoints.* In Ethiopia, each month has 30 days apart from *Pagume*, a month of 5 days just before New Year (in mid-September). Patients were trained to complete the diary by inserting a mark in the appropriate column for that day – either one headed by a picture of a person in bed with ADLA or one headed by a person working in the field (both men and women work in the field in this part of rural Ethiopia). Diaries were collected from the intervention group at every monthly intervention visit. The trial coordinator and CPA supervisor checked back through each diary with the patient using religious days as reminders. Diaries from the control group were collected quarterly and checked in the same way. At each follow-up visit, three month diaries were checked by data collectors and data collection supervisors in the field office before being transferred to data entry. Where discrepancies were observed, data collectors were sent to patients' homes to check dates using religious days.

*Serious Adverse Events.* Serious Adverse Events (SAEs) in both arms were actively elicited by the CPAs and the trial coordinator, and recorded through the CRF each quarter. Reports were confirmed by the trial coordinator and reported within 24 hours to the Local Safety Monitors (LSMs) who reached a consensus with the Principal Investigator on whether the SAE needed classification as a Suspected and Unexpected Serious Adverse Reaction (SUSAR). The Trial Steering Committee reviewed all Serious Adverse Events at each meeting. The investigators and TSC determined that ending the trial early following an interim analysis would be meaningless because the intervention was not available to the population.

*Secondary Outcomes.* Pre-specified secondary outcomes were duration of ADLA, clinical stage of disease, lower leg and foot circumferences, quality of life, disability, stigma, economic productivity, school attendance of household members, and adherence with treatment. A summary measure of the treatment effect was calculated using proportional odds logistic regression models at baseline and 12 months for intervention and control groups. Serious Adverse Events were compared between groups and described by age, gender, diagnosis or cause of death and attribution to the intervention.
Exploratory analysis of the primary endpoint was carried out to examine for effect modification by baseline covariates to determine whether treatment outcome differed between sub-groups or over time. We examined whether treatment outcome differed by sex, by disease severity at baseline or by *kebele* location (distance from the surfaced, all-weather road). For *kebele* location, we created two groups: closer (9 *kebeles*) and remote (9 *kebeles*), and tested for group effect modification on the study outcome using a likelihood ratio test.

Table 1. Outcomes recorded at each visit.

| Baseline | 3 months | 6 months | 9 months | 12 months |
|---|---|---|---|---|
| Socio-demographic | | | | Socio-demographic |
| Economic | | Economic | | Economic |
| ADLA (recall) | ADLA (diary) | ADLA (diary) | ADLA (diary) | ADLA (diary) |
| | SAEs | SAEs | SAEs | SAEs |
| DLQI | DLQI | DLQI | DLQI | DLQI |
| WHO-DAS II | | | | WHO-DAS II |
| Stigma scale | | | | Stigma scale |
| Clinical stage | | Clinical stage | | Clinical stage |
| Mossy lesions | | Mossy lesions | | Mossy lesions |
| Foot & Leg Circumference | | Foot & Leg Circumference | | Foot & Leg Circumference |
| Interdigital lesions | | Interdigital lesions | | Interdigital lesions |

Table 2. Adherence to study intervention among participants randomized to immediate treatment

| Months | Proportion with 100% adherence (seven times a week) N (%) (N=350) | | | | | |
|---|---|---|---|---|---|---|
| | Washing with soap | Applying ointment | Elevation | Exercises | Wearing Socks | Wearing Shoes |
| Month 1 | 230 (66) | 225 (64) | 201 (57) | 221 (63) | 22 (6) | 90 (26) |
| Month 2 | 244 (70) | 243 (69) | 233 (67) | 234 (67) | 19 (5) | 117 (33) |
| Month 3 | 240 (69) | 237 (68) | 220 (63) | 222 (63) | 152 (43) | 190 (54) |
| Month 4 | 310 (89) | 328 (94) | 294 (84) | 289 (82) | 262 (75) | 278 (79) |
| Month 5 | 313 (89) | 308 (88) | 291 (83) | 295 (84) | 257 (73) | 282 (81) |
| Month 6 | 313 (89) | 308 (88) | 291 (83) | 295 (84) | 257 (73) | 282 (81) |
| Month 7 | 301 (86) | 287 (82) | 287 (82) | 285 (82) | 232 (67) | 278 (79) |
| Month 8 | 306 (87) | 301 (86) | 281 (80) | 278 (79) | 236 (68) | 296 (84) |
| Month 9 | 302 (86) | 302 (86) | 298 (85) | 297 (85) | 245 (70) | 286 (82) |
| Month 10 | 299 (85) | 295 (84) | 292 (83) | 291 (83) | 243 (69) | 282 (81) |
| Month 11 | 298 (85) | 300 (86) | 294 (84) | 299 (85) | 260 (74) | 292 (83) |
| Month 12 | 311 (89) | 311 (89) | 306 (87) | 309 (88) | 267 (76) | 300 (86) |

**Table 3. Test for interaction with age (>=50 or <50y)**

```
---------------------------------------------------------------------------
               |                Robust
            _t | Haz. Ratio   Std. Err.      z    P>|z|     [95% Conf. Int]
---------------+-----------------------------------------------------------
Arm (Age <50y) |   .7937592   .061365     -2.99   0.003     .6821547
.9236228
Arm (Age >=50y)|   .8349674   .0495726    -3.04   0.002     .7432469
.9380067

Approx test for unequal RRs (effect modification): chi2(1) =     2.50
                                                    Pr>chi2 =   0.1141
```

**References**

1. Negussie H, Addissie A, Addissie T, Davey G. Preparing for and Executing a Randomised Controlled Trial of Podoconiosis Treatment in Northern Ethiopia: the Utility of Rapid Ethical Assessment. . PLoS Neglected Tropical Diseases 2016;10:e0004531.

2. Joshi D. Validating patients' self-report of acute dermatolymphangioadenitis against health professional diagnosis in northern Ethiopia. [Master Dissertation]. Brighton: Sussex; 2014.

3. Negussie H, Molla M, Fegan G, et al. GoLBeT [Gojjam Lymphoedema Best practice Trial]: description of study protocol for a randomised controlled trial of effectiveness of treatment for podoconiosis (non-filarial elephantiasis) Trials 2015;16:8.

4. Molla YB, Tomczyk S, Amberbir T, Tamiru A, Davey G. Patients' perceptions of podoconiosis causes, prevention and consequences in East and West Gojam, Northern Ethiopia. BMC Public Health 2012;12:828.

5. Sikorski C, Ashine M, Zeleke Z, Davey G. Effectiveness of a Simple Lymphoedema Treatment Regimen in Podoconiosis Management in Southern Ethiopia: One Year Follow-Up. PLoS Neglected Tropical Diseases 2010;4:e902.
